# Lung donation and donor lung management: a survey among health care professionals in Argentina

**DOI:** 10.5935/0103-507X.20210072

**Published:** 2021

**Authors:** Vanesa Romina Ruiz, Sergio Adrián Terrasa, Susana Bauque, Pablo Ezequiel Rodriguez, Verónica Celia Morozovsky, Alejandro Gabriel Da Lozzo, Alejandro Daniel Midley

**Affiliations:** 1 Hospital Italiano de Buenos Aires - Buenos Aires, Argentina.; 2 Fundación Favaloro - Buenos Aires, Argentina.

**Keywords:** Brain death, Health personnel, Tissue and organ procurement, Lung transplantation, Argentina

## Abstract

**Objective:**

To describe health care providers’ knowledge about lung donation and donor lung management.

**Methods:**

A descriptive, cross-sectional study based on an anonymous survey was conducted between March and September 2018 among health care professionals registered to *Sociedad Argentina de Terapia Intensiva*.

**Results:**

Of the 736 respondents, the mean age was 40.5 years (standard deviation 8.9), and 61.3% were female. Sixty percent were physicians, 21.5% were nurses, and 17.9% were physiotherapists. Seventy-eight percent considered themselves appropriately informed about organ procurement, and 79.8% stated that they knew potential organ donor critical care management. The lung donor criteria were answered correctly by 71.3% of the respondents. However, after the donor’s brain death, 51% made no changes to ventilator parameters, 22.9% were not aware of which parameters to reprogram, and 44.5% selected tidal volume of 6 - 8mL/kg and positive end expiratory pressure of 5cmH_2_O. For 85% of the health care providers, the type of apnea test chosen was disconnection from the ventilator, and only 18.5% used a lung management protocol. The most frequent interventions used in the case of arterial oxygen partial pressure/fractional inspired oxygen < 300 were positive end expiratory pressure titration, closed-circuit endotracheal suctioning, and recruitment maneuvers.

**Conclusion:**

Health care professionals surveyed in Argentina correctly answered most of the questions related to lung donor criteria. However, they lacked detailed knowledge about ventilatory settings, ventilatory strategies, and protocols for lung donors. Educational programs are key to optimizing multiorgan donation and should be focused on protecting the donor lungs to increase the numbers of organs available for transplantation.

## INTRODUCTION

Organ procurement is the process of collecting organs and tissues for transplantation to meet the needs of patients on transplant waiting lists.^(^1^)^ A great number of health care professionals (HCPs) take part in this process, both at the in-hospital and out-of-hospital levels. The high demand for transplant organs does not match the number of actual organ donors, and this shortage of organs is the leading cause of death among patients on transplant waiting lists.^(^2^,^3^)^ In Argentina, this situation is compounded by a low organ donation rate, which averages 19.5 donors per million population (DPMP), in comparison to other countries such as Spain (46.9 DPMP).^(^4^,^5^)^

This organ shortage is caused by multiple factors, including legal, economic, technical, medical, cultural, religious, administrative, logistical and educational barriers.^(^3^,^6^,^7^)^ In an international study, surveyed South Americans ranked organ shortage as a critical issue.^(^8^)^ Other studies showed that the HCPs surveyed lacked essential knowledge about donor organ procurement. The main knowledge deficits reported were donor detection, brain death (BD) criteria, and donor management.^(^9^,^10^)^ Furthermore, there is good correlation between organ procurement and transplant rates at hospitals when staff have a good level of education about donation.^(^11^)^ Few studies were published in Latin America, showing great variability in knowledge about the procurement process among professionals in different countries.^(^12^,^13^)^ Several strategies have been proposed in Argentina to increase organ donation rates, but these strategies have not achieved a cultural change in the health care system.^(^1^)^

The first lung transplant in Argentina was performed in 1967. Currently, an average of 24 lung transplants are performed every year.^(^14^)^ Only 15 - 20% of lungs are transplantable due to changes following BD and iatrogenic mechanical ventilation, among others causes.^(^3^,^15^)^ However, the lung procurement rate in Argentina was only 6.7% between 2016 and 2018, which is associated with the country’s very low lung transplantation rate of 0.97 per million population.

In Argentina, there is no consensus among health care centers and regional organ procurement organizations about the management of potential organ donors (PODs).^(^3^,^16^)^ Moreover, ventilatory parameters may be set by the physician and/or nurse or physiotherapist according to the staff available in each center and their level of training. To date, there has been no local research that assesses health care workers’ knowledge about organ donation and their professional experience with multiorgan donor management.

The research questions are as follows: does the lack of knowledge regarding donor management impact the number of donor organ procurements? Do the multiorgan donor management protocols impact donor lung retrieval?

This study aims to describe lung donor knowledge and management among HCPs in Argentina.

## METHODS

### Design

A descriptive, cross-sectional study based on an anonymous survey was conducted between March and September 2018. After identifying and reviewing questionnaires already available in Spanish, which did not cover the range of information required for our study, the authors created a new self-administered instrument, the DonAR survey (Appendix - Supplementary material). This instrument was reviewed by two experts in the field. A pilot test of the instrument was performed at the author´s hospital and the questions were then modified according to the respondents’ feedback. The study was approved by the local Institutional Ethics Committee through resolution 3493. This research was conducted pursuant to the ethical principles described in national and international rules and regulations on human health research, in accordance with regulation 1480/2011 by the Argentine Ministry of Health, the Declaration of Helsinki, the Declaration of Istanbul, and the Good Clinical Practice (GCP) standards issued by the National Institutes of Health. All anonymous data were handled with strict confidentiality. Access was limited to authorized personnel only for research purposes, pursuant to Act 25.326/00 (Argentine Personal Data Protection Act) and Act 26.529/09. Since answering the questionnaire implied active participation, it was assumed that respondents agreed to participate.

### Settings

The *Sociedad Argentina de Terapia Intensiva* (SATI) is a national voluntary professional organization. It includes professionals from across the country who work in adult, pediatric and neonatal critical care units in public or private hospitals and emergency services, both of which are potential areas for POD detection and donor organ procurement in Argentina.

### Population

Health care professionals responsible for the care of PODs in Argentina.

### Sampling

All health care professionals in the SATI database were invited to participate by completing an online questionnaire sent by email. Biochemists, veterinarians and pharmacists were excluded from the study.

### Data collection

The study variables were grouped into four categories: variables related to demographic characteristics - age, gender, profession, speciality, year of graduation from university, organ procurement region, kind of institution and workplace, and ability to diagnose BD in the workplace; variables related to knowledge - level of knowledge, training in organ procurement and/or POD management, definition of BD and detection, reporting and critical care management of PODs, as well as knowledge of donation criteria for lung donors; variables related to professional experience: prior experience with patients with potential BD - apnea testing and types of tests, engagement in POD management and POD protocols; ventilation setting parameters - tidal volume (Vt) and positive end expiratory pressure (PEEP), besides POD ventilation strategy: interventions in cases of low oxygenation and types of strategies used in such cases; types of recruitment maneuvers and PEEP titration; use of closed-circuit endotracheal suctioning and pneumonia prevention measures.

### Data analysis

Categorical variables are shown as numbers and percentages. Continuous variables are presented as the mean and standard deviation (SD) or median and interquartile range, according to their distribution. To determine the distribution of the sample, histograms, skewness and kurtosis parameters, the Shapiro-Wilk test was used. The analysis was performed with Stata 13.0.

### Procedure

The questionnaire could be completed in approximately ten minutes and covered three domains: demographic data; general knowledge about the definition of BD, its diagnosis and lung donor criteria; and professional experience in potential lung donor (PLD) management.

## RESULTS

### Demographic characteristics

A total of 751 completed questionnaires were received, and 15 were excluded (biochemists, veterinarians, and pharmacists). Among the surveyed health care professionals, the mean age was 40.5 years (SD 8.9). Overall, 61.3% were women, and physicians were the most represented professionals (60.6%), particularly physicians working in intensive care (72.2%). Other professionals were nurses (21.5%) and physiotherapists (17.9%), and 78.03% of the latter specialized in respiratory critical care or cardio-respiratory care. Most respondents (74.2%) graduated after 2000, and the percentage of those working at public or private institutions was similar. According to the results, 65.9% worked in adult intensive care units (ICUs), and 16.2% worked in pediatric ICUs. The response rate from the City of Buenos Aires (30.4%) was higher than that from other cities, and among regions, Pampeana had the highest response rate (Table 1). A total of 82.34% of respondents stated that they could give a diagnosis of BD at their workplace.

**Table 1:** Baseline demographic characteristics of the health care professionals surveyed

Variables	Results
Female gender	451 (61.3)
Age (years)	40.5 ± 8.9
Profession	
Physician	446 (60.6)
Nurse	158 (21.5)
Physiotherapist	132 (17.9)
Graduation year	
Before 1980	12 (1.6)
1980 - 1990	61 (8.3)
1990 - 2000	117 (15.9)
2000 - 2010	297 (40.4)
After 2010	249 (33.8)
Distribution by organ procurement region	
Pampeana	381 (51.8)
City of Buenos Aires	224
Province of Buenos Aires	147
La Pampa	10
Center	76 (10.3)
Catamarca	4
Córdoba	64
La Rioja	4
Santiago del Estero	4
**Variables**	**Results**
Cuyo	54 (7.3)
Mendoza	24
San Juan	16
San Luis	14
North Patagonia	53 (7.2)
Neuquén	31
Río Negro	22
Noroeste	50 (6.8)
Jujuy	13
Salta	8
Tucumán	29
Litoral 2	46 (6.3)
Entre Ríos	12
Santa Fe	34
Litoral 1	40 (5.4)
Corrientes	9
Chaco	14
Formosa	9
Misiones	8
South Patagonia	36 (4.9)
Chubut	20
Santa Cruz	7
Tierra del Fuego	9
Workplace	485 (65.9)
Adult intensive care unit	
Pediatric intensive care unit	119 (16.2)
Neonatal intensive care unit	29 (3.9)
Emergency room	43 (5.8)
Operating room	11 (1.5)
Other	49 (6.7)
Kind of institution	
Public	439 (59.7)
Private	297 (40.3)

Results expressed as n (%) or mean ± standard deviation.

### Knowledge

In relation to organ donation, 77.6% of surveyed professionals considered themselves to be appropriately informed. Only 27% of respondents described receiving instruction on organ procurement and/or donor management processes during their graduate studies, and 66.4% described receiving this training during their postgraduate studies.

To 7.2% of the respondents, BD did not imply patient death. A total of 24.3% of respondents identified a neurocritical patient as a POD when the Glasgow Coma Scale was 7 or lower, while 70.5% of respondents considered the patient a potential organ donor only when BD diagnostic criteria were met.

According to the results, 79.8% of surveyed professionals stated that they were familiar with POD critical care management, and 71.3% correctly identified which optimal conditions a PLD must fulfill.

### Management

More than half of the professionals (54.6%) had contacted the *Instituto Nacional Central Único Coordinador de Ablación e Implante* (INCUCAI) to report a patient with potential BD. A total of 97.2% of respondents had had at least one patient with potential BD, and 69.7% (95% confidence interval - 95%CI 64.15 - 71.03) of respondents had participated in the procurement and/or management process of a POD. Of these, 88.8% had performed an apnea test at least once, with the traditional method for apneic oxygenation being the preferred choice. The main findings related to PLD management are detailed in table 2. Only 18.5% of professionals who treated a POD reported having a protocol in their unit to manage a PLD. Most of the professionals who treated a PLD (51%) reported making no changes in ventilation settings. Professionals who did change parameters mainly modified Vt and PEEP. The ventilation strategy chosen by most respondents was a protective strategy, with Vt 6 - 8mL/kg predicted body weight, mostly with a PEEP of 5cmH_2_O. However, 22.9% were not familiar with appropriate parameters to ventilate a PLD.

**Table 2:** Main findings on the health care professional’s management of potential lung donors

Management	n (%)
Apnea test	
Done at least once	442 (88.8)
Type of apnea test	
Preoxygenation and disconnection from the ventilator	378 (85.5)
Artificial increase of CO_2_	30 (6.8)
CPAP	24 (5.6)
Controlled hypoventilation	6 (2.1)
Used a PLD management protocol	92 (18.5)
Ventilation setting after brain death confirmation	
Did not change	244 (49)
Main change in ventilation setting	
Vt and PEEP	138 (56.6)
FiO_2_	64 (26.2)
Other	42 (17.2)
Ventilatory strategy for PLD	
Vt of 6 - 8mL/kg and PEEP of 5cmH_2_O	222 (44.6)
Do not know	114 (22.9)
Vt of 6 - 8mL/kg and PEEP de 8 - 10cmH_2_O	70 (14.0)
Vt of 8 - 10mL/kg	72 (14.4)
Vt of 10 - 12mL/kg	20 (4.1)
Interventions in case of PaO_2_/FiO_2_ < 300 in PLD	
Done at least once	419 (84.1)
Type of interventions	
PEEP titration	268 (64.0)
Endotracheal suctioning	262 (62.5)
Recruitment maneuver	238 (56.7)
Mucus clearance techniques	230 (54.9)
Bronchoscopy	51 (12.2)

CO_2_ - carbon dioxide; CPAP - continuous positive airway pressure; PLD - potential lung donors; Vt - tidal volume; PEEP - positive end expiratory pressure; FiO_2_ - fraction of inspired oxygen; PaO_2_/FiO_2_ - arterial oxygen partial pressure/fractional inspired oxygen.

In the case of a PLD with low oxygenation, defined as the ratio of arterial oxygen partial pressure to fractional inspired oxygen less than < 300, 84.1% of respondents reported making some kind of intervention. The most frequently chosen type of PEEP titration was the PEEP/Compliance Protocol (120/274; 43.8%). The choice of recruitment maneuver was heterogeneous, and the most frequent maneuvers performed were an incremental increase in PEEP to 40cmH2O, followed by an incremental decrease (71/243; 29.2%); a continuous positive airway pressure (CPAP) of 40cmH_2_O for 40 seconds (32/243; 13.2%); and a pressure control-continuous mandatory ventilation mode with an inspiratory pressure of 20cmH_2_O and an incremental increase in PEEP to 20 - 30cmH_2_O (32/243; 13.2%).

Finally, most respondents used closed-circuit endotracheal suctioning (91.8%) and maintained ventilator-associated pneumonia prevention measures.

## DISCUSSION

The main findings of this study were that although most of the professionals surveyed answered lung donor criteria correctly and claimed knowledge of POD management, nearly a quarter did not know which ventilation parameters to select for PLDs, and almost half of them did not choose the recommended ventilatory strategy for PLDs; most used disconnection from the ventilator as an apnea test, and just a few used PLD management protocols.

To address the first research question on HCPs’ knowledge regarding donor management: educational gaps were seen among surveyed professionals. Only a small minority of respondents received information about organ procurement and/or management processes during their graduate studies. Topics that reflected differing knowledge levels included specific aspects of PLD management, although most respondents reported an understanding of POD management. Other studies have shown the same gap in professionals’ education.^(^17^)^ In a literature review by Walters, 58% of papers recommended educating and training HCPs in the organ donation process.^(^18^)^ Lack of communication between the regional procurement agency and health care professionals working in critical care is one of the causes of POD loss.^(^13^)^ In this study, just over half of the respondents contacted INCUCAI. For this reason, it is vital to include organ and/or tissue donation and procurement in the Argentinian university curricula as a means to increase POD detection, actual donor rates and, in turn, increase transplantation rates.^(^8^,^13^)^

An observational survey was performed in 2002 to determine the standard ventilatory and cardiovascular management of POD. The Vt was 10 ± 2mL/kg, the PEEP was 3.3 ± 2.7cmH_2_O, and no changes were made after BD confirmation.^(^19^)^ As well as in the study conducted by Mascia et al.,^(^19^)^ in our study more than half the professionals surveyed did not change ventilatory parameters from a brain protective strategy to a strategy aimed at lung protection following a BD diagnosis. In 2010, the same Italian investigation team compared the effects of a lung protective strategy with a conventional one on the number of lungs available for transplantation. The protective strategy consisted of a Vt of 6 - 8mL/kg of predicted body weight of the POD and a PEEP of 8 - 10cmH_2_O. A closed circuit was used for tracheal suction, apnea tests were performed with the ventilator in CPAP mode, and recruitment maneuvers were performed in cases of disconnection from the ventilator. The number of lungs available for transplantation with the lung protective strategy doubles the number of lungs available following use of the conventional strategy. After this publication, these ventilatory adjustments became international recommendations for POD.^(^20^,^21^)^ However, there was great variability in the choice of ventilation strategy and poor adherence only 14% to the lung protective ventilatory strategy. In addition, almost half of the professionals selected a PEEP of 5cmH_2_O and failed to implement the protocols on PLD management. Furthermore, there are national publications that recommend a protective ventilation strategy as part of PLD management.^(^16^,^22^)^ This situation is worrisome and a possible reason for the low rate of donor lung retrieval in our country. Miñambres et al. compared the donor lung transplantation rate before and after the application of a lung management protocol for POD in Spain. Following a lung protective ventilatory strategy, they doubled lung procurement rates with no apparent differences in early recipient survival.^(^23^)^ In addition to being inexpensive and using technology available in any ICU worldwide, multiorgan donor management protocols have increased lung procurement rates, without having an impact on procurement rates for other organs.^(^21^,^23^,^24^)^

On the other hand, BD certification using ancillary methods is mandatory in Argentina. In the study by Bonetto et al., electroencephalograms and apnea tests were the most common methods used to certify BD.^(^1^)^ Among the surveyed professionals, the most frequently chosen apnea test to certify the irreversible absence of spontaneous respiratory function was apneic oxygenation. However, this technique has some limitations and may contribute to worsening respiratory function in PLDs, as shown by decreases in PaO_2_/FiO_2_, which cause alveolar collapse and, in turn, the loss of lungs viable for transplantation.^(^25^)^ For this reason, current recommendations suggest performing the test in CPAP mode or using a PEEP valve of 10cmH_2_O in the expiratory outlet.^(^16^,^25^,^26^)^ Moreover, some reports indicate that recruitment maneuvers have been used as a single intervention to improve oxygenation or as one more element within a multimodal strategy.^(^21^,^23^,^25^)^ There is no consensus about which recruitment maneuver to perform in PODs. Mascia el al. doubled ventilation with low Vt for 10 breaths in cases of disconnection from the ventilator. Miñambres et al. used controlled ventilation with a PEEP of 18 - 20cmH_2_O for 1 minute, followed by a decrease of 2cmH_2_O each minute, and then an increase of 50% Vt for 10 breaths every hour. Finally, Paries et al. performed the following recruitment maneuver after an apnea test with disconnection from the ventilator: PEEP was increased from 5 to 35cmH_2_O, and the respiratory rate was decreased to 0.5 breaths per minute for 40 seconds.

In Argentina, several strategies have been proposed to increase organ donation: creating the role of hospital coordinators; quality of health care assurance program using the Glasgow Coma Score subprogram; implementing the role of fellow in organ and tissue procurement at the national level; and passing Act 27.447 on Organ, Tissue and Cell Transplantation, which amended the previous Transplantation Act, increasing data collection channels on people’s willingness to donate and introducing presumed consent in cases when the patient had not opted out while still alive. This legislation on organ and tissue transplantation had an immediate impact due to an increase in social awareness, which led to a record level of donors and transplants in 2018. However, the lung usage rate from actual donors has still not increased as expected, and currently, the waiting list for lung transplantation includes 257 patients.^(^1^)^

This study has several strengths. After extensive research on the topic, this study is the first to simultaneously assess knowledge about the procurement process and professional experience in the management of lungs from multiorgan donors. Additionally, this is the first study to include physiotherapists in the survey sample, as physiotherapists are, in some institutions, the ones responsible for ventilatory settings. It is also worth noting the lack of Argentine studies on this topic. For this reason, these results can enrich the academic literature and promote new research on this topic nationally.

This study also has some limitations. Having invited participants based on their inclusion in the SATI database may have resulted in a sample that is not entirely representative of all professionals engaged in this task. On the other hand, data were gathered using an instrument designed by researchers for that specific purpose and that had not been used before. This makes it difficult to compare the results with results from other populations. The response rate was 15.3%, as in other online surveys among HCPs.^(^27^,^28^)^ It is likely that the respondents were the most motivated to report on this subject or the most qualified in the field. Therefore, the authors cannot rule out the fact that results may be influenced by participant selection bias. It can be inferred that the actual knowledge about this topic in the overall population may be even lower than the level recorded in the research, which does not render the results invalid.

The results help us to understand some barriers that may be overcome by changing overall organ procurement processes, especially lung procurement processes, in Argentina. Strategies to mitigate the unmet demand for transplant organs must target health care teams working with neurocritical patients at risk for BD, who can identify, report, and manage potential donors.^(^29^)^ Thus, concerns about donor organ shortages can be turned into actions, and actions into procurement. Despite limitations arising from the size and the selection of the sample, the results of this study revealed that there is a lack of specific knowledge about ventilation strategies as well as an underidentification of POD and a need for PLD management protocols.

## CONCLUSION

Teaching and training health care professionals in Argentina to improve the currently low lung donation rates is key. The challenge is to cultivate a multidisciplinary team that succeeds in treating multiorgan donors with a focus on protecting the lungs to increase donor lung availability for transplantation, thus saving a greater number of lives.
