# Supplementary material for: Lung donation and donor lung management: a survey among health care professionals in Argentina
Source: Rev Bras Ter Intensiva. 2021 Oct-Dec;33(4):557–64. doi: 10.5935/0103-507X.20210072 (PMC8889600; doi:10.5935/0103-507X.20210072)
Supplement: Supplementary file 1 [file rbti-33-04-0557-suppl01.pdf]

Lung donation and donor lung management: a survey among health care professionals in Argentina

Doação de pulmão e manejo do doador: um levantamento entre profissionais de saúde na Argentina

Vanesa Romina Ruiz[1], Sergio Adrián Terrasa[1], Susana Bauque[1], Pablo Ezequiel Rodriguez[1], Verónica Celia Morozovsky[2], Alejandro Gabriel Da Lozzo[1], Alejandro Daniel Midley[1]

Appendix - DonAR survey

DonAR survey

You are invited to participate in the DonAR survey addressed to physicians, nurses and physiotherapists who provide health care services to patients with brain death in critical units in Argentina.

This survey is aim at assessing their knowledge and professional experience towards organ donation during the procurement and management process of potential organ donors.

This survey will take you approximately 10 minutes to complete. You will not benefit from the survey results directly, but they may benefit the community since they will allow researchers to identify possible causes for organ shortage, to evaluate strategies to increase national organ procurement rate, and to educate the health team in the future.

Your participation is optional, which means you will not be compensated and you will not be affected negatively if you decide not to participate. Your answers will be confidential.

By answering this survey, you accept participating in this research. If you have already answered the survey, please disregard this email.

Thank you,

The Research Team

1. Age ____
2. Gender
   ☐ Male
   ☐ Female
3. Profession
   ☐ Physician
   ☐ Nurse
   ☐ Physiotherapist
   ☐ Other
4. You have selected "Physician", please, click in your postgraduate degree
   ☐ Anesthesia
   ☐ Cardiology
   ☐ Thoracic surgery
   ☐ Internal medicine
   ☐ Emergency
   ☐ Neonatology
   ☐ Neurosurgery
   ☐ Neurology
   ☐ Pediatrics
   ☐ Intensive care
   ☐ Other clinical specialty
   ☐ Other surgical specialty
5. You have selected "Physiotherapist", do you have any training in respiratory therapy or cardiorespiratory care?
   ☐ Yes
   ☐ No
6. Please, enter the year in which you graduated ______
7. Please, choose the province where you work
   ☐ Catamarca
   ☐ Ciudad Autónoma de Buenos Aires
   ☐ Córdoba
   ☐ Corrientes
   ☐ Chaco
   ☐ Chubut
   ☐ Entre Ríos
   ☐ Formosa
   ☐ Jujuy
   ☐ La Pampa
   ☐ La Rioja
   ☐ Mendoza
   ☐ Misiones
   ☐ Neuquén
   ☐ Provincia de Buenos Aires
   ☐ Río Negro
   ☐ Salta
   ☐ San Juan
   ☐ San Luis
   ☐ Santa Cruz
   ☐ Santa Fe
   ☐ Santiago del Estero
   ☐ Tierra del Fuego
   ☐ Tucumán

8. In which area do you work?

☐ Adult intensive care unit

☐ Pediatric intensive care unit

☐ Neonatal intensive care unit

☐ Emergency room

☐ Operating room

☐ Other

9. Do you work in a

☐ Public institution

☐ Private institution

10. We know that brain death is a controversial issue. Please feel free to choose the sentence you agree with the most. In regards with level of knowledge about organ donation, you would say that you are

☐ Poorly informed

☐ A little bit informed

☐ Quite informed

11. Did you receive information about organ procurement and management in your graduate studies?

☐ Yes

☐ No

12. And during your postgraduate studies?

☐ Yes

☐ No

13. Do you consider brain death to imply the patient is dead?

☐ Yes

☐ No

14. When do you consider a neurocritical patient as a potential organ donor?

☐ When the patient´s Glasgow Coma Scale is ≤ 7

☐ When lack of brain activity is confirmed with technical or instrumental means in a patient with irreversible absence of cerebral response with loss of consciousness, absence of spontaneous breathing, absence of head reflexes and determination of fixed pupils

☐ When the patient is in a vegetative state

☐ When the patient is in cardiorespiratory arrest

☐ Do not know when to consider a neurocritical patient as a potential organ donor

☐ Other

15. Have you ever contact the Regional Organ Procurement Agency to communicate that you might have a patient with brain death?

☐ Yes

☐ No

16. Do you have the technical means to make the diagnosis of brain death in your workplace?

☐ Yes

☐ No

17. Have you ever worked with a patient who might be brain dead?

☐ Yes

☐ No

18. Have you ever done an apnea test?

☐ Yes

☐ No

19. You have answered that you have done an apnea test. Which test did you do?

☐ Conventional: disconnection from the ventilator with oxygenation

☐ Alternative: artificial increase of CO_2

☐ Alternative: with CPAP

☐ Alternative: with controlled hypoventilation

20. Do you know the appropriate care measures for potential organ donors?

☐ Yes

☐ No

21. Have you ever participated actively in an organ procurement or management process?

☐ Yes

☐ No

22. You have answered that you have participated actively in an organ procurement or management process. Now we are going to ask you about lung management of the potential organ donor. Which of the following is the optimal lung donor criteria?

☐ $PaO_2 > 300$ on FiO_2 1.0, PEEP 10cmH_2O, no evidence of aspiration/sepsis, sputum *Gram* stain - absence of organism, absence of purulent secretions at bronchoscopy

☐ Chest radiograph with minimal infiltrates, no evidence of aspiration/sepsis, no prior cardiopulmonary surgery, sputum gram stain - absence of organism, absence of purulent secretions at bronchoscopy

☐ Clear chest radiograph, $PaO_2 > 300$ on FiO_2, PEEP 5cmH_2O, absence of chest trauma, no evidence of aspiration/sepsis, no prior cardiopulmonary surgery, sputum *Gram* stain -absence of organism, absence of purulent secretions at bronchoscopy

☐ Unilateral chest trauma, no evidence of aspiration/sepsis, no prior cardiopulmonary surgery, sputum *Gram* stain - absence of organism, absence of purulent secretions a bronchoscopy

☐ Do not know the optimal criteria for lung donors

23. Is there a lung management protocol in your workplace?

☐ Yes

☐ No

☐ Do not know

24. Do you make any changes in the ventilatory parameters after the diagnosis of brain death in a potential lung donor?

☐ Yes

☐ No

25. Which are the main changes that you make in the ventilatory parameters after the diagnosis of brain death in a potential lung donor?

☐ Change of FiO_2

☐ Change in minute volume (tidal volume and respiratory rate)

☐ Change in tidal volume and PEEP

☐ Other

26. Which parameters do you select to ventilate a potential lung donor?

☐ Tidal Volume 10 - 12mL/kg, PEEP 5 cmH_2O

☐ Tidal Volume 10 - 12mL/kg, PEEP 8 - 10cmH_2O

☐ Tidal Volume 8 - 10mL/kg, PEEP 5cmH_2O

☐ Tidal Volume 8 - 10mL/kg, PEEP 8 - 10cmH_2O

☐ Tidal Volume 6 - 8mL/kg, PEEP 5cmH_2O

☐ Tidal Volume 6 - 8mL/kg, PEEP 8 - 10cmH_2O

☐ Do not know the ventilatory parameters to ventilate a potential lung donor.

☐ Other

27. If a potential lung donor has low oxygenation ($PaO2/FiO2 < 300$), do you consider making any intervention?

☐ Yes

☐ No

28. You have selected to make an intervention if a lung donor has low oxygenation. Which are the most used strategies?

☐ Semi lateral decubitus

☐ Mucus clearance techniques

☐ Suction endotracheal tube

☐ Bronchoscopy

☐ Recruitment maneuvers

☐ PEEP titration

☐ Other

29. If you use recruitment maneuvers, please answer the next question. Otherwise click on “Next”. Click on the most used recruitment maneuver in a lung potential donor

☐ Sight with increases of tidal volume during several breaths

☐ CPAP of 40cmH_2O during 40 seconds

☐ CPAP of 30cmH_2O during 30 seconds

☐ Increasing PEEP until 40cmH_2O and then decreasing step by step

☐ Pressure control, continuous mechanical ventilation with an inspiratory pressure of 25 - 30cmH_2O and PEEP 10 - 15cmH_2O during two hours for one time only

☐ Pressure control, continuous mechanical ventilation with an inspiratory pressure of 30cmH_2O and increasing PEEP until 20 - 30cmH_2O

☐ Controlled ventilation with PEEP of 18 - 20cmH_2O for 1 minute and decreasing by 2cmH_2O each minute, then increasing 50% tidal volume for 10 breaths

☐ Other

30. If you use PEEP titration, please answer the next question. Otherwise click on “Next”. Click on the most used PEEP titration in a lung potential donor

☐ PEEP/FiO_2 table (ARDS Network)

☐ PEEP / Compliance Protocol

☐ Determination of the lower inflection point by pressure / volume curve

☐ PEEP increase until plateau pressure $<$ 28cmH2O (Express Protocol)

☐ Esophageal manometry guided

☐ Guided by pulmonary ultrasound

☐ Guided by volumetric capnography

☐ Other

31. Do you use a closed-circuit for endotracheal suctioning?

☐ Yes

☐ No

32. Do you think that the care bundle to prevent ventilator-associated pneumonia (such as head elevation, endotracheal balloon control, pharinks suctioning, etc.) should continue after brain death confirmation?

☐ Yes

☐ No
